# Responses to Developmental Temperature Fluctuation in Life History Traits of Five Drosophila Species (Diptera: Drosophilidae) from Different Thermal Niches

**DOI:** 10.3390/insects12100925

**Published:** 2021-10-11

**Authors:** Tommaso Manenti, Anders Kjærsgaard, Toke Munk Schou, Cino Pertoldi, Neda N. Moghadam, Volker Loeschcke

**Affiliations:** 1 Department of Biology, Aarhus University, Ny Munkegade 114-116, DK-8000 Aarhus, Denmark; andersk@clin.au.dk (A.K.); tokeschou@gmail.com (T.M.S.); volker@bios.au.dk (V.L.); 2 Laboratori Biokyma srl, Loc.Mocaia 44b, 52031 Anghiari, AR, Italy; 3 Section of Biology and Environmental Science, Aalborg University, Frederik Bajers vej 7H, DK-9220 Aalborg, Denmark; cp@bio.aau.dk (C.P.); neda.nasiri@gmail.com (N.N.M.); 4 Aalborg Zoo, Mølleparkvej 63, DK-9000 Aalborg, Denmark; 5 Department of Biological and Environmental Science, University of Jyväskylä, FI-40014 Jyväskylä, Finland

**Keywords:** thermal physiology, fluctuating temperature, Jensen’s inequality, temperature variance, acclimation, wing size, climate change, developmental time, viability, wing aspect ratio

## Abstract

**Simple Summary:**

Most laboratory experiments on insects to date have been conducted using constant temperature settings. Even when the purpose of the study was to investigate effects of temperature, insects have mostly been kept at different but constant temperatures ignoring natural variation in temperature. Here we investigated effects of simple daily temperature fluctuation (22.5/27.5 °C and 20/30 °C) on some development characteristics in five species of fruit flies (*Drosophila*) originating from areas with different temperature profiles. We demonstrated how species of the same genus can show substantial differences when developing at fluctuating temperatures not always predictable by development at comparable constant temperature (25 °C).

**Abstract:**

Temperature has profound effects on biochemical processes as suggested by the extensive variation in performance of organisms across temperatures. Nonetheless, the use of fluctuating temperature (FT) regimes in laboratory experiments compared to constant temperature (CT) regimes is still mainly applied in studies of model organisms. We investigated how two amplitudes of developmental temperature fluctuation (22.5/27.5 °C and 20/30 °C, 12/12 h) affected several fitness-related traits in five Drosophila species with markedly different thermal resistance. Egg-to-adult viability did not change much with temperature except in the cold-adapted *D. immigrans*. Developmental time increased with FT among all species compared to the same mean CT. The impact of FT on wing size was quite diverse among species. Whereas wing size decreased quasi-linearly with CT in all species, there were large qualitative differences with FT. Changes in wing aspect ratio due to FT were large compared to the other traits and presumably a consequence of thermal stress. These results demonstrate that species of the same genus but with different thermal resistance can show substantial differences in responses to fluctuating developmental temperatures not predictable by constant developmental temperatures. Testing multiple traits facilitated the interpretation of responses to FT in a broader context.

## 1. Introduction

The physiology, behavior, and life history of ectothermic organisms is highly affected by the ambient temperature because of its influence on biochemical processes [1]. As a consequence, temperature is an important driver of macro-ecological processes such as species distributions and habitat choice [2,3]. Numerous studies have demonstrated clinal genetic variation in thermal tolerance correlated with local mean temperatures and latitude or altitude within species [4,5,6] and responses to global warming such as shifts in phenology now occur widely [7,8,9].

Given the large impact of ambient temperature on all levels of biological organization of ectotherms it is important that ecologically relevant temperatures are used in experiments investigating thermal performance. Nevertheless, studies that use fluctuating temperatures, either to directly address their effects on the phenotype or indirectly to obtain natural phenotypes to be used in a different context, have been underrepresented until recently [10,11,12,13,14,15,16,17,18]. This in spite of the fact that some extent of daily temperature fluctuations are the norm.

One of the complications of using fluctuating temperature (FT) regimes in experiments relates to the non-linear nature of thermal performance of ectotherms. Performance usually increases only slowly at low (tolerable) temperatures followed by an accelerating and then linear increase in the intermediate temperature range before it levels off around the maximal performance. Beyond the temperature where performance is maximal, it decreases sharply with higher temperature [19,20,21]. Therefore, phenotypic traits influenced by FT are not equal to the arithmetic mean temperature if steady state performance over the temperature range is non-linear. However, if the shape of the thermal norm of reaction (e.g., thermal performance curve) derived from steady state estimates at representative temperatures along the curve and the frequency distribution are known, the performance with FT can potentially be predicted with Jensen’s inequality [22,23]. Basically, this mathematical property of non-linear functions states that variance around the mean of the independent variable will consistently elevate or depress the mean response of the dependent variable. If a function is accelerating (second derivative is positive), variance will result in an elevated response whereas if the function is decelerating (second derivative is negative) variance will depress the response [23]. Although Jensen’s inequality can be helpful in predicting responses to temperature variance, biological rate processes are affected by previous environmental conditions. Thus, Jensen’s inequality will not take into account that changes in the body temperature can lead to acclimation responses via changes in, e.g., enzymatic activity (1) or cell membrane properties [24,25]. Plastic acclimatory responses can, however, be costly [26]. The benefits of invoking a plastic response therefore depend on how fitness is integrated across environments.

Theory predicts that plastic responses are affected by organism’s experiences with spatial or temporal environmental change (i.e., within the lifespan or developmental stage of an individual) and predictability. A fixed or canalized phenotype that performs reasonably well over a range of environmental temperatures is expected to evolve [27,28,29]. However, a specialist may also evolve by restriction of activity to certain favorable environments. Evolution in stable environments with only minor environmental fluctuations should also lead to specialists that perform well at the predominant thermal conditions compared to generalists, but which show a relatively rapid decrease in performance as temperature departs from the prevailing thermal conditions due to antagonistic pleiotropy [30,31,32]. Even if thermal heterogeneity is expected to shape plastic responses, evolutionary studies hardly confirm this hypothesis [33]. A large plastic response was observed when a non-laboratory adapted population of *D. simulans* was exposed to predictable and unpredictable fluctuating thermal regimes during development and the early adult stage showed in all life history and stress resistance traits investigated [34]. However, contrary to the expectation of the authors, twenty generations of laboratory thermal selection in constant, predictable or unpredictable thermal fluctuating regimes did not affect the plastic response although evolutionary changes in trait mean performance was observed in all traits investigated [35].

The main objective of this study was to investigate if the plastic responses to developmental temperature fluctuation in the model organism *D. melanogaster* (Meigen) and in other four species of the same genus with different evolutionary trajectories and thermal resistance. We expected that species presumably differing in developmental acclimation capacities by adaptation to different thermal niches would lead to species specific patterns in response to developmental temperature fluctuation (small amplitude FT: 22.5/27.5 °C and large amplitude FT: 20/30 °C, 12/12 h). We tested several fitness-related developmental traits (egg-to-adult viability, developmental time, wing size, and wing aspect ratio) and compared trait responses from FT regimes to CT development at the mean temperature (25 °C). We also included the two extreme temperatures experienced in the high fluctuation treatment (20 and 30 °C) as CTs to estimate the shape of the reaction norm for the traits and the expected responses with fluctuation in the absence of developmental acclimation effects.

On the basis of the temperature conditions at the collection sites of the species (Table S1) and available information, we predicted that *D. melanogaster*, which is a widespread generalist species, would perform reasonably well in all environments [36,37,38]. Therefore, the response to temperature fluctuation should mirror the expectations based on the shape of the reaction norm obtained from the CT treatments. This species is adapted to adverse environmental conditions and some degree of unpredictability removing the basis for, and the value of, adaptive phenotypic plasticity. This is further exacerbated by gene flow preventing, to some degree, local adaptation. However, if temperature stress occurs with high developmental temperature, depressed trait values with large FT but not small amplitude FT are expected due to costs associated with stress responses. *D. immigrans* (Sturtevant) is a cold adapted widespread species [39]. This is reflected in the temperature conditions of the collection site (Table S1), so we expected that the high temperature (constant or periodically) would be detrimental in terms of relative fitness. In contrast, the cactophilic *D. buzzatii* (Patterson and Wheeler) is adapted to high temperatures usually within the range tested here or even higher so we expected this species to be well adapted to our FT regime behaving according to expectations from the CT performance curve. The desert species, *D. mojavensis* (Patterson), is also adapted to high temperatures living in columnar cacti in the Sonoran desert, south-western North America [40,41], so we also expected this species to be tolerant to FT in the form of diurnal temperature shifts. The last species included in this study, *D. bipectinata* (Duda), is a tropical rainforest species experiencing relatively little diurnal and annual temperature variation [41]. The temperature profile at the collection site of this species has the smallest variance in mean temperature (Table S1). We therefore expected negative effects on relative fitness with FT. Not so much because of the temperature range tested but because rapid temperature shifts are rarely encountered and therefore expected to induce stress responses conceptually similar to stress responses to unpredictable environmental changes which are usually more severe than with predictable changes [34,42,43].

## 2. Materials and Methods

### 2.1. Experimental Setup

The fly populations of the Drosophila species we used here (*D. melanogaster*, *D. immigrans, D. buzzatii, D. mojavensis, D. bipectinata*) were the same as those used in Kellermann et al. [44]. Collection sites and temperature data for each locality is given in Table S1. For each species 30 adult flies were transferred from the mass rearing bottles to each of 30 vials and provided a plastic spoon filled with standard oatmeal-sugar-yeast-agar medium and sprinkled with live yeast for egg laying. The age of the flies laying eggs was species specific but represented the first few days of reproductive maturity for the individual species to avoid aging effects. After three hours of egg-laying the spoons were removed and the eggs collected with a flattened preparation needle. Twenty eggs were transferred to each of twenty vials containing 7 mL standard oatmeal-sugar-yeast-agar medium for each temperature treatment giving a total of 400 eggs per treatment per species. Due to special dietary requirements of the cactophilic *D. mojavensis*, a banana Opuntia medium was used for this species [44].

### 2.2. Temperature Regimes

The treatment temperatures were 20, 25, and 30 °C (±1 °C) as constant temperature (CT) and two fluctuating temperatures (FT), 22.5/27.5 °C and 20/30 °C (small and large amplitude FT), alternating between the two temperatures as a step function every 12 h in phase with a 12/12 light/dark cycle common to all treatments. The transient temperature changes lasted 30 min for both treatments (22.5/27.5 °C and 20/30 °C). Hence, the mean of the fluctuating temperatures was 25 °C and the large amplitude FT treatment fluctuated between the low and high constant temperature treatments (20 and 30 °C). This temperature range is often used in Drosophila studies and, although many Drosophila species can develop at lower temperatures, the difference in development- and growth rates below 20 °C is much smaller than corresponding differences at higher temperatures as discussed above. The cabinets had an internal volume of approximately 400 L. The cabinets were equipped with a thermal isolated case and two internal units to heat or cool down the air temperature and to ensure airflow and temperature homogeneity. A computer with in-house-designed software controlled the temperature and the light in the cabinets. The temperature inside each cabinet was also recorded by a datalogger placed inside an empty vial of the same size of those that hosted the flies to verify the right temperature. Before running the experiment the cabinets were calibrated for three month in order to minimize the differences between average temperatures to less than 0.3 °C. The racks with vials were randomly redistributed within each climate cabinet twice daily to account for possible slight temperature heterogeneities in the cabinets.

### 2.3. Phenotypic Traits

Developmental time was scored twice daily (at 10:00 and 22:00) and calculated in hours from the midpoint of the three hours egg laying interval. Emerging flies were transferred to Eppendorf tubes and immediately frozen for morphometric measurements and scoring of egg-to-adult viability. The wings of three males and three females from each respective vial were removed and placed on microscope slides with a droplet of acetic acid giving a maximal sample size of 60 wing pairs per sex and temperature treatment. The wings were photographed digitally using a camera attached to a dissecting microscope and a computer with the software IM1000 version 1.1. Measurements of wing vein landmark positions were obtained with the software tpsDig2 version 2.16 [45]. Ten landmarks were digitized as shown in Figure S1.

### 2.4. Statistical Analyses

All analyses were conducted with R statistical software version 3.3.0 (The R Foundation for Statistical Computing 2016). Egg-to-adult viability (arcsine square root transformation of the ratio of emerged adults to transferred eggs), wing size (measured as mean centroid size of the right and left wing), and wing aspect ratio (measured as the mean distance between landmarks 1 and 4 in Figure S1 divided by wing length estimated as the distance between landmarks 2 and 9 of the right and left wing) were analyzed with conventional analysis of variance (ANOVA). Separate analyses were conducted for the CT treatments (20, 25, and 30 °C) and the different FT treatments with the same mean (25 °C, 22.5/27.5 °C and 20/30 °C). Except for egg-to-adult viability, species and sex were entered as fixed effects and vial nested in temperature and species entered as a random effect. Sexes were pooled for egg-to-adult viability as a preliminary analysis of the sex ratios as a function of temperature came out non-significant (results not shown). Before analysis all traits were standardized by subtracting the mean and dividing by the standard deviation to facilitate comparisons between the species [46]. We conducted within species analyses for all traits to get a more detailed picture of the species specific effects of temperature treatment and sex. Development time was not formally analyzed due to the nature of the data (collected in regular intervals and decreasing variance with temperature). Instead these data were visualized by bootstrapping the temperature specific point estimates and 95% confidence intervals (1000 replicates).

## 3. Results

### 3.1. Constant Temperature Comparisons

**Figure 1:**
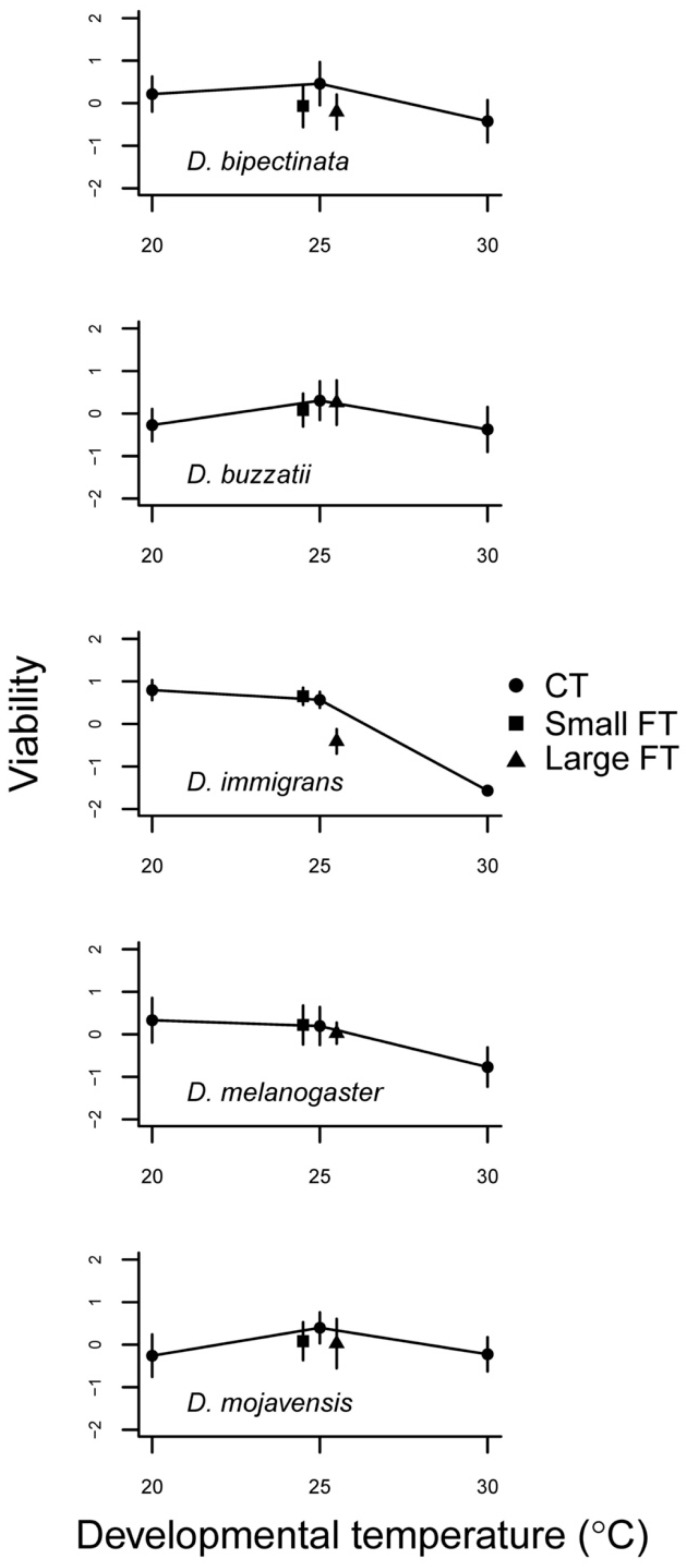
Mean egg-to-adult viability of the combined sexes as a function of developmental temperature regimes in five Drosophila species. Error bars represent 95% confidence intervals. Data were arcsine square root transformed and scaled (see text). CT: Constant developmental temperature; Small FT: Small amplitude fluctuating developmental temperature; Large FT: Large amplitude fluctuating developmental temperature.

**Figure 2:**
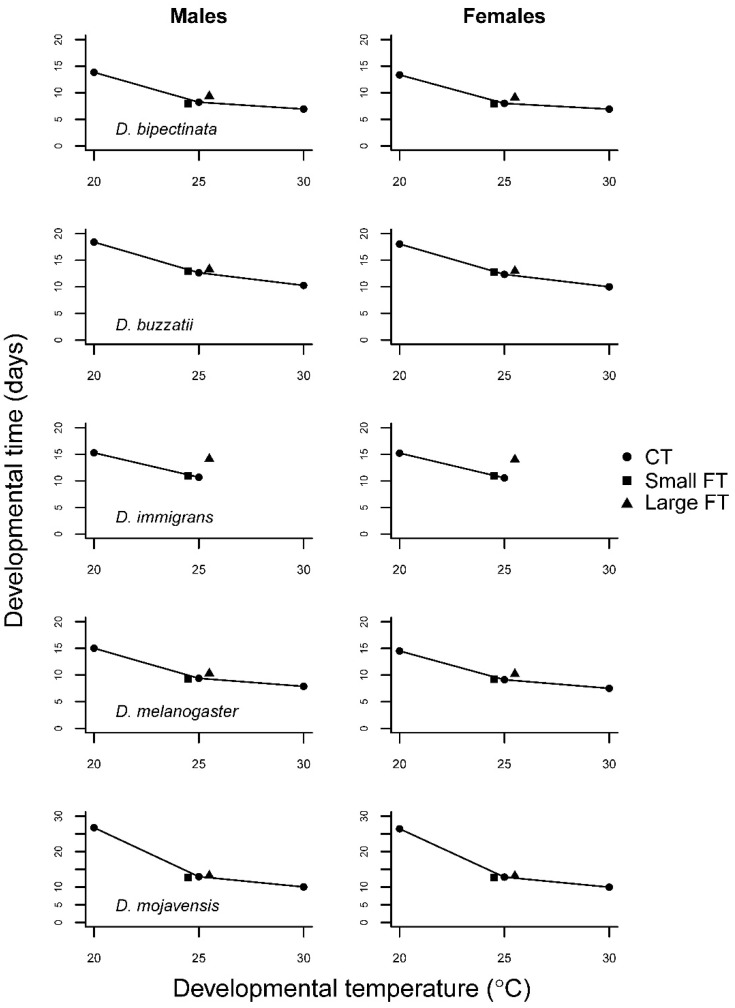
Mean developmental time in days as a function of developmental temperature regime in five *Drosophila* species. The error bars are not visible because they are hidden behind symbols.

All species had high egg-to-adult viability at 25 °C (Table 1 and Table S2, Figure 1). At 20 °C, the two heat adapted species *D. buzzatii* and *D. mojavensis* had lower viability. At 30 °C, all species showed declining viability to different degrees, the most affected being *D. immigrans* with none surviving followed by *D. melanogaster* and *D. bipectinata*. Developmental time decreased with temperature as expected. The largest drop was seen going from 20 °C to 25 °C and most pronounced in *D. mojavensis* (Figure 2).

**Figure 3:**
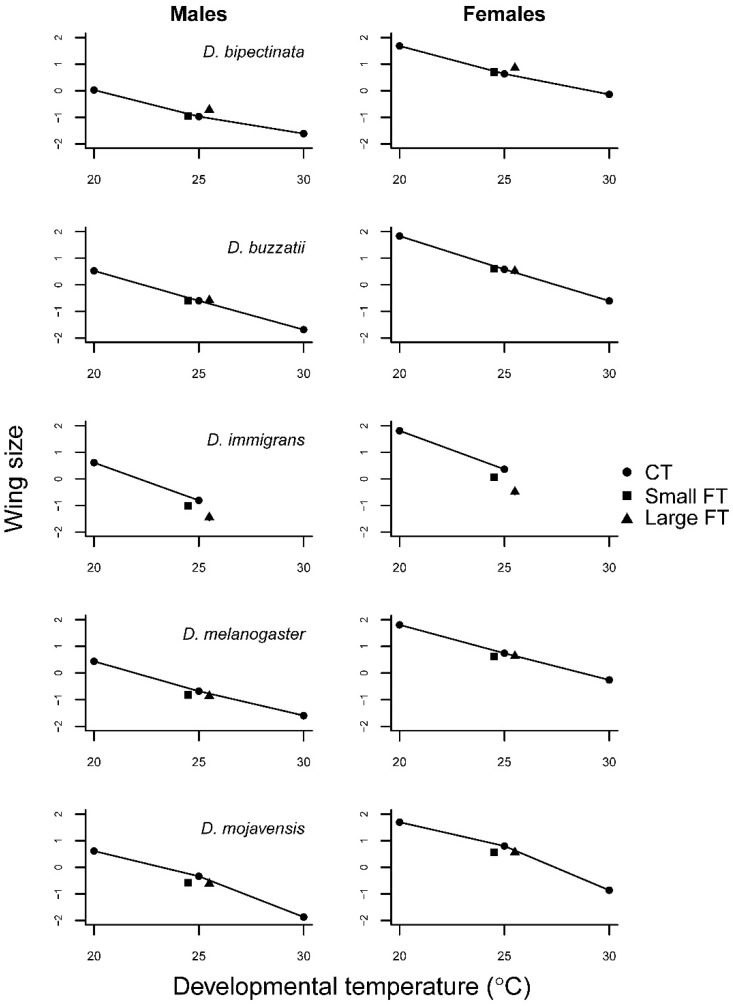
Mean wing size as a function of developmental temperature regimes in five Drosophila species with 95% confidence intervals. The error bars are not visible because they are hidden behind symbols.

**Figure 4:**
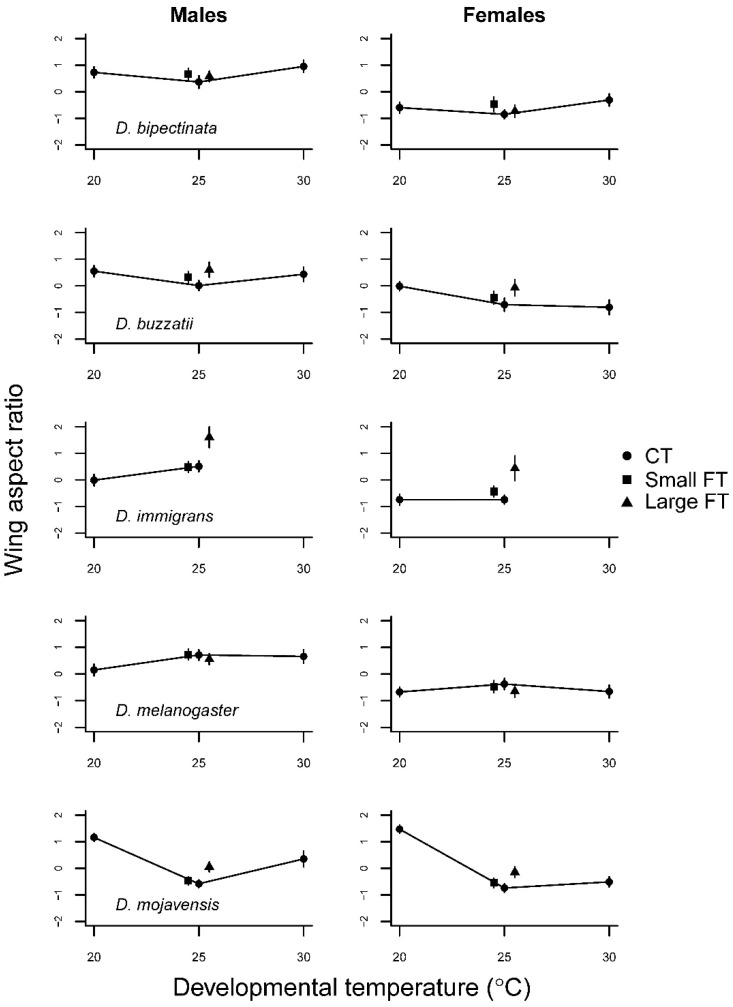
Wing aspect ratio (wing width divided by wing length) as a function of developmental temperature regimes in five *Drosophila* species with 95% confidence intervals.

Wing size decreased in a quasi-linear fashion with temperature in all five species (Figure 3, Table S3). A temperature by sex interaction was observed in *D. bipectinata* and *D. buzzatii* (Table 2). Wing aspect ratio was highly variable between species (F4,1238 = 1195.9, *p* < 0.001), temperature (F2,1238 = 59.0, *p* < 0.001), and sex (F1,1238 = 530.5, *p* < 0.001) (Table S4). Males generally had higher wing aspect ratios than females, i.e., relatively broader wings. (Figure 4). In *D. bipectinata*, the wing aspect ratio accelerated with temperature whereas in the other species a concave or decelerating (both positive and negative) thermal performance curve was observed.

### 3.2. Fluctuating Temperature Comparisons

Only egg-to-adult viability of *D. immigrans* was affected by temperature variance (Figure 1; Table 1). However, whereas none survived in the high CT treatment, substantial numbers survived the periodical exposure to high temperature in the FT treatment. Developmental time in response to FT regime seemed to be affected in all species but *D. mojavensis* (Figure 2). This was remarkable in light of the large effect of low CT on developmental time in this species resulting in a concave reaction norm.

There were clear qualitative differences in the impact of fluctuating temperatures on wing size between species. In the separate analyses for each species no effect on the wing size of either sex due to temperature variance was found for *D. buzzatii* (Table 2; Figure 3). *D. bipectinata* showed a positive linear (females) or accelerating (males) increase in wing size with increasing FT, whereas the opposite was the case for *D. immigrans* (Figure 3). *D. melanogaster* and *D. mojavensis* displayed an effect when going from constant 25 °C to small fluctuations, but only little or no change with larger fluctuation (Figure 3, Table 2). Wing aspect ratio mostly increased, i.e., wings became relatively broader with temperature fluctuations (Figure 4), but the responses were sex (Table 2) and species specific (Table S4). The effect of temperature fluctuations was remarkably larger for wing aspect ratio than in most of the other investigated traits sometimes matching or even exceeding the differences in effect sizes of the CTs going from 25 °C to 20 °C or 30 °C (in female *D. bipectinata, D. buzzatii,* and *D. immigrans*; Figure 4).

## 4. Discussion

We compared several developmental traits in five Drosophila species from different thermal niches in response to three constant developmental temperatures (CTs) and two fluctuating developmental temperatures (FTs) with the same mean. We found that responses differed substantially in both CT and FT regimes between species and across the investigated traits. Moreover, the trait responses to temperature fluctuations did not always mirror the expectations based on the CT performance curves suggesting that developmental effects altered trait values in ways that were species and trait specific.

Egg-to-adult viability should be highly canalized across all but the most stressful developmental temperature conditions as this trait is, by definition, closely related to fitness [47,48]. All species were affected by the high CT, albeit *D. buzzatii* only marginally so, indicating that development at 30 °C was stressful for all species. However, transient exposure to this temperature for 12 h daily did not give rise to a significant effect on egg-to adult viability compared to the same mean CT 25 °C except in the very heat sensitive *D. immigrans* and at the boundary of significance in *D. bipectinata* (Table 1). The latter species was interestingly predicted to be sensitive to temperature fluctuation. Based on [44], the upper thermal limits for these two species varied between 36.5 °C and 36.9 °C and between 38.2 °C and 38.4 °C, respectively. For these the latter species was interestingly predicted to be sensitive to the daily exposure to 30 °C more than to daily rapid change of temperature fluctuation. In the two heat adapted species *D. buzzatii* and *D. mojavensis* both the high and low CTs appeared stressful compared to 25 °C but the effect was small and temperature fluctuations consequently had little or no effect (Figure 1; Table 1). Thus, egg-to-adult viability was, as expected, highly canalized in response to temperature fluctuations where periods of less stressful conditions could effectively prevent mortality [49,50].

Although egg-to-adult viability was largely unaffected by FT and transiently stressful conditions, we can expect fitness-consequences to be realized in other traits if canalization of survival is costly. For example, stress responses to high temperature such as activation of conditionally expressed heat shock proteins have been shown to affect other traits such as growth, fertility, and fecundity, negatively [51,52]. The expression of temperature sensitive alleles of other genes will also play a significant role in how FT influences quantitative traits. Especially if governed by antagonistic pleiotropy, i.e., when an allele is beneficial in one environment but detrimental in another [30,31,32,53].

The traits developmental time and body size (here estimated crudely by wing size, see [54]) potentially allow for some flexibility, in terms of energy uptake and allocation, to canalize the expression of the most important traits. They are intricately related in the determination of the mean growth rate of juvenile insects [47,55]. Deviations in these traits compared to CT development therefore likely reflect trade-offs with one another or with survival directly. In the widespread and widely used model species, *D. melanogaster* developmental time with large amplitude FT was prolonged relative to 25 °C CT but not with small amplitude FT. This is in line with the expectation based on the CT performance curve, although we cannot strictly evaluate trait expression with small amplitude FT because we did not include the corresponding CTs (22.5 and 27.5 °C). Unlike developmental time, wing size, was affected by even small amplitude FT despite a linearly decreasing CT performance curve (Figure 3). The wing size of *D. melanogaster* therefore appears to be sensitive to FT in a manner not predictable by the CT performance curve. In line with what Manenti et al. [34] found, a small wing size when flies are tested in FT suggests that developmental temperature acclimation responses were in play also within the more benign part of the temperature range.

In the other species tested, developmental times with FT were qualitatively similar to that in *D. melanogaster* with little effect of small amplitude FT but a large effect of large amplitude FT (Figure 2). The effect of FT was smallest in *D. buzzatii* as expected from the linear performance curve. Wing size in this species was, likewise, highly canalized not deviating from the CT which collectively suggests that it does not rely on inducible stress defenses in this temperature range. In contrast, the other heat adapted species, *D. mojavensis*, showed a remarkably longer developmental time at 20 °C than at the higher CTs, which, however, did not affect developmental time with FT compared to 25 °C CT but it was much faster than expected. Development thus appears to be arrested at night when temperature drops significantly in its natural desert environment. This pattern was reiterated in wing size which was smaller with both small and large amplitude FT suggesting a larger influence of the high temperature. *D. bipectinata*, which was expected to be sensitive to FT due to the relatively modest temperature variation in its natural tropical environment, increased markedly in wing size with large amplitude FT. This response was surprising, although the performance curve was slightly accelerating towards the lower temperature and the response was thus in the direction predicted by Jensen’s inequality. In contrast, D. melanogaster, in particular, had similar CT performance curves but smaller wing size with FT. Assuming that wing size is reasonably correlated with body size [56] similar results have been found in other studies for this species [10,34,57,58,59]. A possible explanation for the apparent abnormal response in *D. bipectinata* could be that it is a specialist adapted to a narrow range of temperatures and therefore does not rely on inducible defense mechanisms that may be costly to maintain (26). Consequently, more energy is allocated to growth. The potential cost is seen in the lower egg-to-adult viability with FT compared to the other species as indicated by the higher F test statistic (F = 2.4 vs. F ≤ 0.7; Table 2) with the exception of *D. immigrans*. Furthermore, although not tested here, we could expect to see a higher degree of developmental instability [57,60,61] due to insufficient inducible repair mechanisms.

The effect of FT on wing aspect ratio has rarely been investigated [62], although changes in wing aspect ratio with temperature can be adaptive by optimizing flight to a commonly experienced temperature range [63]. We are, however, inclined to see the changes in wing aspect ratio observed in this study as incapacity to canalize wing aspect ratio for two reasons. First, the phenotypic variance, which is a commonly used as an indication of developmental instability [60], is generally higher with high CT and high amplitude FT for wing aspect ratio (Figure 4). Second, high CT and high amplitude FT usually affected other trait values negatively and we mostly see changes in wing aspect ratio with these temperature regimes. The fact that wing aspect ratio changes were mostly large compared to the effects of FT seen in the other traits suggests that wing aspect ratio is a sensitive indicator of temperature stress [64]. Apart from the wing aspect ratio, only weak temperature by sex interactions were observed suggesting that the sexes share a common genetic basis for thermal tolerance during development as suggested by Williams et al. [65] investigating heat knock down performance in *D. melanogaster*.

From the data presented here it is clear that using FT regimes in experiments can alter trait values and thereby possibly affect or change the conclusions that are drawn from, often less ecologically relevant, CT regimes with the same mean temperature. This is especially problematic when comparing several species with different capacity for phenotypic plasticity and canalization when facing FTs. Furthermore, the use of extreme CTs in assessments of thermal adaptation and responses to global warming likely overestimate the stressful effects of temperature because transient periods at more benign temperatures are effective in minimizing the effects on egg-to-adult viability as observed elsewhere for the sibling species *D. melanogaster* and *D. simulans* [10,50]. Conversely, at benign CTs, effects of temperature stress on trait values may be underestimated because inducible stress defenses are not activated and antagonistic pleiotropy is not in play. Small amplitude FT can, however, also be beneficial because high but non-stressful developmental temperature accelerates growth and promotes acclimation to stress later in life [13,66,67,68]. At the very least such considerations should be taken into account when designing experiments involving multiple species and generally in the interpretation of data [13,68]. In this study a step function was used for alternating between the high and low temperature settings which is a simplification of naturally occurring diurnal temperature shifts. We, however, believe that even a relatively simple approach such as this is a significant improvement in terms of ecological relevance away from the standard constant temperature treatments for most ectotherm species.

When multiple species are compared, observed trait responses may be influenced by phylogenetic relatedness of the species, e.g., Pagel [69]. In the present study there does not seem to be a strong phylogenetic signal. The two species most closely related (*D. buzzatii* and *D. mojavensis*) show quite distinct trait responses in spite of inhabiting similar and well-defined thermal niches among the species tested in the study. It cannot be ruled out that part of the observed trait responses can be explained by inbreeding or laboratory adaptation. However, as argued by Kellermann et al. [70], effects of laboratory adaptation and inbreeding are likely to be considerably smaller than the species effects in interspecies comparisons. Their test of a subset 19 outbred species in their study revealed only minor effects on stress resistance. Furthermore, Kristensen et al. [71] found no effects of inbreeding on the capacity for developmental acclimation to cold resistance testing multiple Drosophila species. In our experiment, the perhaps most distinct result was obtained for the developmental time of *D. mojavensis* at low constant temperature. Although this species was obtained from a stock centre we note that this response mirrors what has been found independently elsewhere on freshly caught populations. Namely that this species develops very slowly at temperatures below 22–23 °C [72].

In conclusion, our predictions were mostly confirmed showing that knowledge about environmental characteristics such as the temperature variance and temperature stress are useful in predicting responses to global warming. This is important to keep in mind because, just as the mean temperature is rising, so is the variance in temperature in many geographic regions [73,74,75,76]. It is also important to underline that there can be substantial differences in trait values depending on the nature of the fluctuations. Unpredictable temperature changes have been shown to be more stressful than comparable but more predictable FT regimes such as regular day/night cycles under some circumstances [34,42,43]. The results of our study may thus be seen as conservative estimates of the effects of FT because the regular fluctuations we used may allow for predictable regulation of gene expression thus representing a form of steady state.

## Figures and Tables

**Table 1:** Effects of temperature on egg-to-adult viability within species. Separate tests were conducted for the constant (20, 25, and 30 °C) and fluctuating temperature (25 °C CT, small FT and large FT) comparisons. Error DF in parenthesis.

Populations	Constant Temperature Viability			Fluctuating Temperature Viability		
Species	DF	MS	*F*	DF	MS	*F*
*D. bipectinata*	2 (54)	3.82	4.0 *	2 (55)	2.37	2.4
*D. buzzatii*	2 (57)	2.67	2.8	2 (57)	0.27	0.3
*D. immigrans*	2 (57)	67.95	243.8 ***	2 (56)	6.87	28.5 ***
*D. melanogaster*	2 (57)	7.21	6.9 **	2 (57)	0.22	0.3
*D. mojavensis*	2 (56)	2.63	3.2 *	2 (56)	0.77	0.7

*: *p* < 0.05, **: *p* < 0.01, ***: *p* < 0.001.

**Table 2:** Effects of temperature and sex on wing size and wing aspect ratio within species. Separate tests were conducted for the constant (20, 25, and 30 °C) and fluctuating temperature (25 °C CT, small and large amplitude FT) comparisons.

Wing Size							
		Constant Temperature			Fluctuating Temperature		
Species	Factor	DF	MS	*F*	DF	MS	*F*
*D. bipectinata*	Temperature	2	9.27	1775.6 ***	2	0.21	41.7 ***
	Sex	1	24.09	4617.2 ***	1	24.41	4862.8 ***
	Temp × sex	2	0.03	5.1 **	2	0.01	1.18
	Error DF	237			243		
*D. buzzatii*	Temperature	2	17.13	2248.4 ***	2	0.004	0.4
	Sex	1	14.43	1893.8 ***	1	12.24	1382.0 ***
	Temp × sex	2	0.04	5.3 **	2	0.01	0.9
	Error DF	277			266		
*D. immigrans*	Temperature	1	37.14	2836.6 ***	2	2.87	191.0 ***
	Sex	1	26.69	2037.8 ***	1	28.85	1923.0 ***
	Temp × sex	1	0.00	0.3	2	0.06	4.3
	Error DF	162			198		
*D. melanogaster*	Temperature	2	21.94	1776.3 ***	2	0.13	13.8 ***
	Sex	1	28.76	2328.4 ***	1	33.26	3416.1 ***
	Temp × sex	2	0.01	0.9	2	0.01	0.6
	Error DF		277		280		
*D. mojavensis*	Temperature	2	19.86	3741.2 ***	2	0.26	60.2 ***
	Sex	1	9.72	1831.1 ***	1	12.33	2888.2 ***
	Temp × sex	2	0.01	2.4	2	0.001	0.3
	Error DF	281			281		
Wing aspect ratio							
*D. bipectinata*	Temperature	2	4.56	16.2 ***	2	1.69	5.1 **
	Sex	1	72.44	256.4 ***	1	67.98	205.0 ***
	Temp × sex	2	0.05	0.2	2	0.11	0.3
	Error DF	238			244		
*D. buzzatii*	Temperature	2	3.32	17.7 ***	2	2.81	14.9 ***
	Sex	1	15.95	85.2 ***	1	12.20	64.6 ***
	Temp × sex	2	0.99	5.29 **	2	0.02	0.1
	Error DF	277			266		
*D. immigrans*	Temperature	1	0.91	7.0 **	2	5.97	44.9 ***
	Sex	1	14.82	113.1 ***	1	23.3	175.5 ***
	Temp × sex	1	0.98	7.5 **	2	0.22	1.99 ***
	Error DF	163			199		
*D. melanogaster*	Temperature	2	2.50	8.6 ***	2	0.68	2.3 ^†^
	Sex	1	47.49	162.3 ***	1	54.82	188.3 ***
	Temp × sex	2	0.81	2.8 ^†^	2	0.06	0.2
	Error DF	278			279		
*D. mojavensis*	Temperature	2	24.12	250.6 ***	2	2.40	32.0 ***
	Sex	1	1.23	12.8 ***	1	0.41	5.5 *
	Temp × sex	2	2.02	21.0 ***	2	0.02	0.3
	Error DF	282			281		

^†^: *p* < 0.10, *: *p* < 0.05, **: *p* < 0.01, ***: *p* < 0.001.

## Data Availability

Data will be made publicly available before publication.

## Supplementary Materials

The following are available online at https://www.mdpi.com/article/10.3390/insects12100925/s1, Figure S1: title: the ten wing landmarks used to calculate wing size and shape; Table S1: collection site (collection time), latitude, annual mean temperature (MT), mean of daily low (TMIN), and high (TMAX) temperature (weatherbase.com, accessed on 10/10/2021). Table S2: results of the analysis comparing egg-to-adult viability of species and developmental temperature. Separate tests conducted for the constant (20, 25, and 30 °C) and fluctuating (25 °C CT, small and large amplitude FT) temperature regimes. Table S3: Results of the analysis of wing size comparing species, temperatures and sex. Separate tests conducted for the constant (20, 25, and 30 °C) and fluctuating (25 °C CT, small and large amplitude FT) temperature regimes. Table S4: Results of the analysis of wing aspect ratio comparing species, temperatures and sex. Separate tests conducted for the constant (20, 25, and 30 °C) and fluctuating (25 °C CT, small and large amplitude FT) temperature regimes.
