# Supplementary material for: Responses to Developmental Temperature Fluctuation in Life History Traits of Five Drosophila Species (Diptera: Drosophilidae) from Different Thermal Niches
Source: Insects. 2021 Oct 11;12(10):925. doi: 10.3390/insects12100925 (PMC8540664; doi:10.3390/insects12100925)
Supplement: Supplementary file 1 [file insects-12-00925-s001.zip › insects-1421366-supplementary.pdf]

insects MDPI

Supplemental material

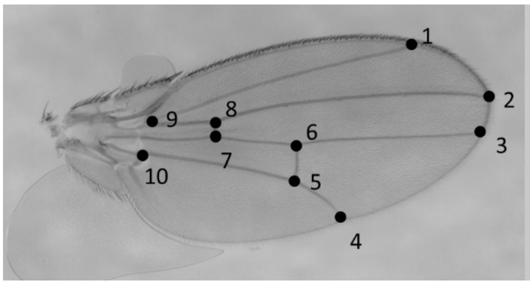

Figure S1. The ten wing landmarks used to calculate wing size and shape.

Table S1. Collection site (collection time), latitude, annual mean temperature (MT), mean of daily low (T_{MIN}) and high (T_{MAX}) temperature (weatherbase.com). The experiment was conducted in November 2010.

Species	Collection site	Latitude	MT	T_{MIN}	T_{MAX}
D. bipectinata	Gordonvale, Queensland, Australia (Feb. 2008)	16.55 S	24.1 ± 2.61	20.1 ± 2.73	29.1 ± 2.33
D. buzzati	Chumbicha, Catamarca, Argentina (2009)	28.26. S	20.3 ± 5.31	13.4 ± 5.82	27.7 ± 4.85
D. immigrans	Aarhus, Denmark (2009)	56.18 N	8.6 ± 5.42*	4.0 ± 4.71**	10.8 ± 6.94
D. melanogaster	Melbourne, Vic., Australia (Dec. 2007)	37.41 S	14.3 ± 4.16	8.9 ± 3.00	19.0 ± 5.08
D. mojavensis	Anza Borrego, CA, USA	33.13 N	22.6 ± 7.30	14.3 ± 6.68	30.9 ± 7.95

*No data available for January and February. Mean biased upwards.** No data available for December. Mean biased upwards.

Table S2. Results of the analysis comparing egg-to-adult viability of species and developmental temperature. Separate tests conducted for the constant (20, 25 and 30°C) and fluctuating (25°C CT, small FT and large FT) temperature regimes.

	Factor	DF	MS	*F*
Constant Temperature	Temperature	2	18.65	70.42***
	Species	4	42.83	161.72***
	Temperature × species	8	5.59	21.13***
Fluctuating temperature	Temperature	2	1.83	6.20**
	Species	4	30.05	101.98***
	Temperature × species	8	2.07	7.03***

**: $P < 0.01$, ***: $P < 0.001$

Table S3. Results of the analysis of wing size comparing species, temperatures and sex. Separate tests conducted for the constant (20, 25 and 30°C) and fluctuating (25°C CT, small FT and large FT) temperature regimes.

	Constant temperature			Fluctuating temperature		
Factor	DF	MS	F	DF	MS	F
Temperature	2	354.0	21037.6***	2	11.58	1402.6***
Species	4	321.3	38193.4***	4	302.85	36671.9***
Sex	1	88.2	10480.6***	1	90.12	10912.6***
Temperature × species	7	1.8	211.3***	8	0.70	84.6***
Temperature × sex	2	0.3	33.6***	2	0.02	2.7
Species × sex	4	1.4	165.4***	4	1.41	170.4***
Temp. × species × sex	7	0.0	0.9	8	0.02	2.4*

*: $P < 0.05$, ***: $P < 0.001$

Table S4. Results of the analysis of wing aspect ratio comparing species, temperatures and sex. Separate tests conducted for the constant (20, 25 and 30°C) and fluctuating (25°C CT, small FT and large FT) temperature regimes.

	Constant temperature			Fluctuating temperature		
Factor	DF	MS	F	DF	MS	F
Temperature	2	11.86	59.0***	2	10.68	52.1***
Species	4	240.42	1195.9***	4	267.62	1306.7***
Sex	1	106.65	530.5***	1	108.04	527.5***
Temperature × species	7	8.24	41.01***	8	2.20	10.7***
Temperature × sex	2	2.62	13.1***	2	0.13	0.6
Species × sex	4	8.72	43.4***	4	9.76	47.7***
Temp. × species × sex	7	0.39	1.9	8	0.09	0.4

***: $P < 0.001$
